# Individuals’ perceptions of the factors linked to everyday soft drink consumption among university students: qualitative study

**DOI:** 10.3389/fnut.2024.1388918

**Published:** 2024-08-19

**Authors:** Mohammed Mahmoud Sarhan, Shemaa Adel Aljohani, Yara Ahmad Alnazzawi, Nuorah Awadh Alharbi, Shihanah Eid Alotaibi, Ahmed Saad Alhujaili, Maram Ali M. Alwadi

**Affiliations:** ^1^Department of Preventive Dental Sciences, College of Dentistry, Taibah University, Al-Madinah al-Munawwarah, Saudi Arabia; ^2^College of Dentistry and Hospital, Taibah University, Al-Madinah al-Munawwarah, Saudi Arabia; ^3^Department of Dental Health, College of Applied Medical Sciences, King Saud University, Riyadh, Saudi Arabia

**Keywords:** nutrition, health, environment, health promotion, diet

## Abstract

**Background:**

Soft drink consumption is continuing to grow worldwide, posing an increasing threat to people’s health and general wellbeing. Consequently, we must understand the factors driving soft drink consumption to support improvements to nutrition. This paper adopts a qualitative research approach to explore individuals’ perceptions of the factors linked to daily soft drink consumption among university students in Saudi Arabia.

**Materials and methods:**

This research employed purposive sampling to recruit a total of 19 students attending university in Al Madinah Province, Saudi Arabia, all of whom reported that they had previously consumed soft drinks. Over 3 months, data was gathered through a mixture of online and in-person semi-structured interviews. Once completed, the interviews were then transcribed and analyzed using inductive thematic analysis to identify the themes that emerged from the data.

**Results:**

This study reveals five core themes: taste, habit, price, environment and social context, and health concerns. Regarding health concerns, this study finds that the public’s increasing concerns about health can reduce people’s consumption of soft drinks. Significantly, this research reveals that the rise in health concerns among the public is being driven by the growing conversation about healthy food and the negative impact of consuming sugary soft drinks occurring in wider society.

**Conclusion:**

To conclude, this research underlines the value of adopting a holistic approach to promoting healthier drink choices (and thus reducing soft drink consumption). Interventions that focus on factors associated with soft drink consumption, such as habits, price, environment, social settings, health concerns, and taste, will be better able to decrease soft drink consumption and improve people’s nutritional intake.

## 1 Introduction

Over the last 10 years, soft drinks have seen a substantial rise in sales and have become a key category in the beverages industry (1). A “soft drink” is defined as a cold beverage that is non-alcoholic, such as lemonade, fizzy drink, or fruit juice (2). The sugar content of soft drinks varies with some high in sugar and others, sweetened with low-calorie artificial sweeteners, advertised as low in sugar, or even “sugar-free.” For many people, soft drinks are the main source of their daily sugar intake (2). Yet, soft drink consumption can have severe consequences for people’s oral and general health.

The impact of soft drink consumption is wide-ranging and is associated with negative outcomes for people’s general wellbeing (3–6). Previous studies have repeatedly underlined the positive link between the regular consumption of soft drinks and an increased risk of type 2 diabetes (5, 6). Furthermore, soft drinks can have adverse outcomes for people’s oral health, with studies showing a positive correlation between soft drink consumption and dental problems including erosion and caries (7, 8).

People across the globe consume soft drinks (9–12). To illustrate, in Australia, 14% of adolescents regularly drink at least four cups of soft drinks weekly (9). Children in Northeastern China drink around 2214.04 ± 2188.62 mL of soft drinks ever week (11). Turning to the adult population in Saudi Arabia, a significant 67% of Saudi Arabian adults report drinking soft drinks every week, highlighting the popularity of this type of drink in the country (12). Clearly, soft drinks are a key component of diets globally.

A variety of factors impact the consumption of soft drinks in different countries and for different demographics (9, 10, 13–18). Consumption is driven and shaped by factors including taste, price, convenience, and availability (9, 10, 15). Typically, people choose to consume drinks that are tasty, affordable, and easy to access. Consumption patterns are shaped by preferences around flavor as well as specific drink types (13). Moreover, soft drink consumption is increased when they are consumed with meals (13). Social settings where soft drinks are present and social influences such as those of a family or peer group also impact soft drink consumption (13), as do promotions, ready availability and other environmental cues (14). Importantly, people’s behaviors around soft drinks are also shaped by the growing understanding of the health impact of soft drinks and targeted taxation (16).

Studies conducted in Saudi Arabia have examined similar factors to those listed above to explore soft drink consumption patterns in the country (19–21). To illustrate, one paper (19) concerning Saudi adults reports two key groups of contributing factors, namely, individual factors including the use of a TV or other electronic device and eating at home, and socio-environmental factors including social events, affordability, availability, and advertising. Research among Saudi Arabian girls of school age reveals that factors such as taste, the soft drink consumption behavior of parents and availability help shape this demographic’s soft drink consumption (20). Longer time spent watching TV has also been found to be linked to higher consumption of soft drinks (21). This body of cross-sectional research offers worthwhile findings regarding the consumption of soft drinks but it also highlights the need for qualitative research to more deeply explore the factors impacting consumption. In response, this research aims to explore individuals’ perceptions of the factors that influence the everyday soft drink intake of university students in Saudi Arabia. A qualitative research design can offer a more detailed understanding of the contextual elements, perceptions, and factors that impact people’s behaviors around soft drinks. This research can then help to inform more effective interventions aimed at reducing the consumption of soft drink across the population and thus improve overall nutritional health in society.

## 2 Methods

### 2.1 Study design

This research employs a qualitative research methodology for data collection and analysis.

### 2.2 Study context

The research participants were undergraduate university students recruited from one governmental university in Al Madinah Province, Saudi Arabia. Madinah Region or, alternately, Al Madinah Province, is a region of the Kingdom of Saudi Arabia (KSA) consisting of 13 territories (22). The capital city of this region is Medina or Al Madinah and the province is found along the Red Sea coastline in the west of the country (22). In total, Al Madinah Province covers 149,207 square kilometers and, in 2017, recorded a population in the range of 2.1 million (22). The Ministry of Education is the chief body responsible for education in Al Madinah Province (23). The region is home to approximately 733 girls schools and 724 boys schools. One particularly well-regarded school is Taibah High School (23).

There are a number of respected higher education institutions in Al Madinah Province. A key educational hub is Taibah University, which has 16 colleges across the region (24). Another important institution is Industrial College, which was established in 1989 and attracts international students alongside students from all across KSA (23). Moreover, in 1998, the Al Madinah College of Technology was created (25). This institute provides a range of degrees in STEM subjects, including mechanical technology, computer technology, electronic technology, electrical technology, administrative technology, and more (25). Al Madinah Province is a culturally rich region, as well as a leader in education and, as such, is making a meaningful contribution to KSA’s development (23).

### 2.3 Sampling strategies

This research employed purposive sampling to recruit students currently completing a bachelor’s degree at a specific university in Al Madinah Province, Saudi Arabia. The students all reported that they drank soft drinks. This research defines a “soft drink” as a cold, flavored, sugar-sweetened non-alcoholic carbonated drink. Potential participants were directly approached by the researchers at their colleges. In simpler words, the research team divided themselves to visit various colleges and searched for individuals who showed interest in participating in the study. Once they identified potential participants, they approached them and explained the purpose of the study. After the participants expressed their initial acceptance, their contact details were collected. The data saturation point was reached in this study, as described by Ritchie and Spencer (26), where no new themes or data were revealed by the interviewees.

### 2.4 Study participants

This research recruited 11 female and eight male students as participants (19 participants in total). The participants were all from seven colleges within the university and were aged from 18 to 25.

### 2.5 Data collection methods

Nineteen semi-structured interviews were conducted in this study to collect the data required. Part of the interviews were completed online, while another part involved approaching individuals face-to-face during their break time for in-person interviews. The average length of each interview for one participant is 23 min. Each study participant was interviewed once. The primary language spoken in the interviews was Arabic but the participants had the choice of using Arabic or English. The interviews were conducted over 3 months starting in September 2023 and ending in December of the same year.

### 2.6 Data collection instrument

Zoom was chosen by the participants to conduct the online interviews. The researchers prepared a topic guide and questions for these interviews drawing on their academic experience and a review of the existing literature. The goal of the guide was to make the interview topic clear to the participants and encourage free, insightful conversations about the factors associated with soft drink consumption. The guide included several open-ended questions designed to allow the interviewees to discuss any experiences or thoughts they considered pertinent. The research team was ready to benefit from any feedback given by the research participants about how to improve the interview questions during the data collection process, but no feedback was provided. The research team was also committed to ensuring that the participants fully understood the interview questions, which they achieved by asking the participants during the interviews whether they understood the question they had just been asked. The topic guide questions were as follows:

1. Can you please describe your general everyday drinking habits?
2. Can you please give a few examples of situations that drove you to drink a soft drink or stopped you from drinking one?
3. Why do you drink soft drinks?
4. What does having a soft drink mean to you?

To ensure that all of the interviewees’ responses were captured accurately, the audio from all the interviews was recorded.

### 2.7 Data processing

The researchers transcribed the audio recordings of the interviews in Arabic and then translated these transcripts into English.

### 2.8 Data analysis

The researchers collectively decided on the data analysis strategy and implemented this strategy from the first semi-structured interview. Specifically, the researchers decided to employ thematic analysis by following the steps described by Braun and Clarke (27–29). First, the researchers became familiar with the data; then, they established the first codes; next, they identified any emerging themes; they then reviewed the themes and refined them; next, they gave the final themes names; finally, they wrote a report on the research results.

To familiarize themselves with the data, each researcher read the interview transcripts and took notes of any data that could potentially address the research aim. After this review, the researchers created an initial set of codes to describe the patterns revealed by the data. Next, the researchers grouped these codes by theme and worked to refine the codes. The themes identified were repeatedly reviewed and different themes emerged gradually throughout the analysis. During the review and analysis of the final interview transcripts, no new themes emerged. Specifically, after reviewing the transcript of Participant 19, the researchers determined that data saturation had been reached and no further data needed to be collected. Each of the themes identified was given a brief name that concisely described the subject of the theme. As the final step, the researchers composed the study report describing the themes identified.

According to Braun and Clarke (27), to ensure their data analysis follows a clear direction, researchers must stick to each of the steps of thematic analysis. These steps enable research teams to define a theme and what it involves, decide whether theoretical or inductive analysis is preferable, determine whether to select a constructionist or realist approach to the thematic analysis and, finally, decide whether the themes the researchers have identified are latent or semantic.

In line with Braun and Clarke (27), the themes identified in this research are considered to reflect the meanings of the data collected. Nonetheless, the frequency with which a theme appears in the data is not necessarily seen as a reflection of its importance, as per Braun and Clarke (27) and Nowell et al. (30). This research uses thematic analysis where themes are derived directly from the data collected and are not pre-prepared based on a literature review of the existing thinking. As a result, a realist thematic approach is adopted in this research as the collected data is considered to be a true reflection of the study participants’ experiences and not simply the outcomes of any cultural or social drivers. Moreover, given that the themes are considered to reflect the direct meanings of the data, the themes identified in this study are semantic rather than latent. This research does not explore factors external to the direct meanings of the data gathered from participants, for example, any cultural and social influences that could have impacted the participants’ interview answers.

### 2.9 Confirming the credibility and trustworthiness of the data analysis

To confirm the credibility and trustworthiness of the data analysis conducted in this research, member-checking and an audit trail were used. In particular, an audit trail was created to establish a record of the decision-making of the researchers, as well as the processes employed during the data gathering and analysis stages. Additionally, the use of member-checking ensured the accuracy and completeness of the researchers interpretations and the results of the study.

### 2.10 Ethical considerations

The participants’ rights and privacy were safeguarded for the entirety of this study. The researchers sought and received ethical approval for this research from Taibah University, College of Dentistry Research Ethics Committee (TUCDREC/110923). From start to end, the anonymity and confidentiality of the participants were protected. In particular, the participants were allocated pseudonyms. Prior to data collection, the participants were given an information sheet on the study and encouraged to ask any questions about the study they may have had. All participants were educated about the study before then giving their written consent to act as participants. As per O’Brien et al. (31), the research report resulting from this study follows the relevant qualitative research reporting guidelines.

## 3 Results

Five key themes were identified from the analysis of the transcripts of the interviews conducted with 11 female and eight male students. The interviewees were all from seven colleges within the one university and aged from 18 to 25. The key themes identified were taste, habit, price, environment and social context, and health concerns.

### 3.1 Taste

Taste emerged as a core theme from the analysis of the qualitative data collected to explore the factors linked to soft drink consumption. The participants repeatedly expressed that taste is a key driving force behind their decision to consume soft drinks. The delicious taste of soft drinks was, in turn, linked to their fizziness and sweetness. Also, the participants stressed that this delicious taste was not offered by any other beverage and made soft drinks unique and unable to be substituted. The following responses reveal the general feeling of the participants regarding the irreplaceable, desirable taste of soft drinks:

“I usually consume soft drinks and for me, the reason why I drink them is the taste. The taste, I love sugar, there is no substitute for soft drinks.” (P 11)

“I tried to cut down on soft drinks, but I start drinking them again for the taste. You cannot compare them with other drinks as sometimes I do not like the taste of other drinks.” (P 2)

“I consume soft drinks for the taste. I love the fizziness.” (P 10)

### 3.2 Habit

A further theme identified from the data analysis was habit and its role in influencing the consumption of soft drinks among the participants. The data shows that habit plays an important role in shaping the participants’ soft drink consumption behaviors. In particular, the participants stress how soft drinks have become a part of their daily eating habits. The participants described how they typically drink a soft drink with meals and viewed this drink as a fundamental part of their meals. The routine of drinking a soft drink with meals is captured in the following responses:

“You know, I get used to consuming soft drinks, it’s a kind of habit to drink them, I enjoy them. They are an essential part of my daily meals.” (P 1)

“I consume soft drinks daily with lunch. I feel it’s impossible to eat without soft drinks.” (P 6)

### 3.3 Price

A noteworthy finding revealed by the qualitative data is that the current “regular” pricing of soft drinks did not impact the participants’ consumption behavior. The participants repeatedly explained that the regular prices of soft drinks were not a significant consideration for them when deciding to buy soft drinks. Rather, the participants saw the price of these drinks as straightforward and in line with their perceived value. The following quotations reveal that price is not generally considered by the participants when they decide to buy a soft drink:

“Also, the price of soft drinks is normal. I can afford it easily, I do not think about it.” (P 2)

“The price of soft drinks is very fair. I think everyone can buy them.” (P 14)

### 3.4 Environment and social context

Surrounding environments and social contexts were repeatedly highlighted by the participants as considerations affecting their soft drink consumption. The data shows that specific settings have a substantial impact on soft drink consumption. In particular, social events with friends were found to encourage soft drink consumption, as did eating in restaurants, and soft drink consumption also rose on weekends. According to the participants, they drank more soft drinks in certain contexts because of their greater availability. In particular, the data shows that soft drinks are easily accessed in shops and restaurants. The participants often referred to the impact of their environment and social context when discussing their soft drink behavior, as in the following examples:

“Usually, I consume soft drinks when I go out with friends, when I eat at restaurants and on the weekends.” (P 9)

“When I am home and no soft drinks are available, I do not go out to buy them. I drink more when I’m out, when I eat at restaurants. This is because you can always find a shop that sells soft drinks and 100% of the restaurants offer soft drinks.” (P 16)

### 3.5 Health concerns

In terms of the factors that are negatively impacting soft drink consumption, health concerns emerged as a significant factor that is negatively correlated with consumption. The qualitative data shows that the participants were acutely aware of the potential adverse health effects of soft drink consumption due to the drinks’ high sugar content. Greater awareness of health risks had inspired the participants to think again about how many soft drinks they consumed. The participants explained that they had developed a greater awareness of health issues from a variety of sources, most notably social media. The following quotations show how health concerns were influencing the soft drink consumption behavior of the participants:

“Sometimes, I drink too much soft drink and sometimes I reduce how much I drink, I control my consumption to take care of myself as they contain a lot of sugar. I want to take care of my health.” (P 5)

“For one period in my life, I completely cut down on soft drinks for 1 year, when I started to regularly play football and joined a gym. They are not healthy, they are all sugar. I knew about their side effects and I developed this awareness from my gym trainer and social media.” (P 7)

## 4 Discussion

Based on the researchers review of the literature, this research represents the first qualitative study to examine individuals’ perceptions of the factors linked to everyday soft drink consumption among university students conducted in the context of Saudi Arabia. The research results identify several core themes or factors that impact people’s soft drink consumption behavior. First is taste, which is a significant driver of consumption. According to the participants, the enjoyable taste of soft drinks is centered on its fizziness and sweetness. Next, habit emerged as a theme, whereby consuming soft drinks had become a part of people’s daily dietary routines. In particular, they are drunk automatically with meals. Given that the participants considered soft drinks to be inexpensive, price was found to play no part in the decision-making around their soft drink consumption. In contrast, the surrounding environment and social contexts were found to play a key role in influencing the participants’ soft drink consumption, with social events, eating in restaurants and weekends encouraging increased consumption. Finally, the qualitative data reveals that health concerns are also affecting how many soft drinks people consume. The participants explained that their increased awareness of the potential negative health outcomes of drinking sugary drinks such as soft drinks was affecting their attitudes toward their consumption. Specifically, health concerns had encouraged them to re-examine their decision to frequently drink soft drinks.

A great deal of research has found that taste is an important driver of soft drink consumption (10, 14, 15). One paper (10) found that taste is the number one reason why people choose soft drinks over other drink choices. This finding was confirmed by further research (15) that highlighted soft drinks’ essential characteristics, with taste emerging as a primary driver of consumption. The present research reaffirms these findings but adds further insights into the important factor of taste. Namely, this research finds that it is the sweetness and fizziness characteristics of soft drinks that are the fundamental elements of their pleasing taste. Moreover, this research finds that the participants considered these features to be unique to soft drinks and not found in other beverages. Thus, on the taste level, there are no suitable substitutes for soft drinks. Typically, soft drinks contain sugar, and a large body of research asserts that sugar can appeal to consumers because it has addictive characteristics (32, 33). Therefore, it is argued that the use of sugar to attract consumers can result in habitual sugar consumption and, sometimes, addiction to sugar and sugary products (33). Nonetheless, Westwater et al. (34) argue that there is insufficient evidence to conclude that sugar is addictive and thus people do not necessarily become addicted to sugar.

The impact of habit on soft drink consumption has also been explored by several studies (16, 17). Research conducted by Zoellner et al. (16) identified habit as a driver of soft drink consumption, while research by Krukowski (17) similarly found that habit is a contributing factor of soft drink consumption when the participants of this study noticed how their soft drink consumption changed in line with changes to their daily routines. This study develops these findings by looking more closely at the habits that affect soft drink intake. The participants of this study highlighted their habit of drinking a soft drink with their meals and their perspective that soft drinks were an essential, accepted part of meals. The habit of automatically drinking a soft drink particularly with a meal has been examined in the existing research (13), which shows that people consume soft drinks with meals to better enjoy the meal.

In relation to soft drinks (15, 17, 19) and the more general context of fast food (35–38), financial components and pricing have been comprehensively researched and examined as potential contributing factors of consumption. According to one report (15), selling soft drinks in a large, inexpensive can increase people’s soft drink consumption. This research confirms these results by finding that participants do not consider the factor of price when deciding whether to purchase a soft drink as they consider soft drinks to be extremely affordable. This suggests that imposing a tax on soft drinks to bring up the price could reduce consumption. In their study, Briggs et al. (39) found that a 20% tax on sugary drinks could help to reduce obesity, especially among people under 30 who are the biggest consumers of soft drinks. Further research (17) has revealed that there is a popular belief that a tax would reduce people’s soft drink consumption. Some of the participants of this study reported that a 50–100% tax would stop them from drinking soft drinks altogether (17). In terms of a 20% tax, the responses of the participants differed with some saying it would have a deterrent effect and others saying it would not reduce their soft drink consumption (17).

The impact of environmental factors on people’s consumption of soft drinks is another topic that has been widely researched (10, 14, 16, 17, 19). Nonetheless, what constitutes “the environment” is broad and difficult to define and several sub-categories have emerged in the studies, with researchers exploring many different aspects of our environment and certain results overlapping. To illustrate, work by Hattersley et al. (14) finds that the factors of availability and advertising can increase the consumption of soft drinks. Krukowski (17), in contrast, explores the environment of the home and finds that concerns about soft drink storage and availability can shape consumption habits. Other work (19) notes that socio-environmental elements including social events, affordability, availability and advertising can contribute to the consumption of soft drinks. This research enhances our understanding of these factors by identifying that soft drink availability, especially in restaurants and shops, significantly contributes to soft drink consumption.

Despite the varying definitions of what constitutes “the environment,” the research done in this area universally emphasizes the influence of environmental factors on the consumption of soft drinks. The health promotion guidelines provided in the Ottawa Charter by the World Health Organization, Regional Office for Europe (40) and Watt’s (41) recommendations all recognize the important impact our environment has on our behaviors. Typically, environmental factors are viewed at the “material” level whereby, as per Shove et al. (42) and Vihalemm et al. (43), changing materials is vital to establishing healthy behaviors in society. Yet, other literature argues that making changes at the “meanings” level (42–44) is as important to reducing unhealthy practices, such as soft drink consumption. To illustrate, regarding soft drink consumption, interventions should create new meanings for different drinks that dissuade the public from consuming soft drinks and encourage them to make healthier choices. So, if one of the meanings of soft drinks is currently “enjoyed as part of a meal,” the goal of targeted interventions should be to attach this meaning to another, healthier type of drink. Changing the meanings attached to soft drink consumption can persuade people to pick healthier options that they can still enjoy and find refreshing.

The impact of health concerns and the increasing health awareness on soft drink consumption has been the subject of several studies (14, 15, 17). Research (14) has found, for instance, that people have a general awareness of the health risks of consuming high volumes of caloric beverages. Additionally, health-related beliefs have been found to shape, to various extents, peoples’ drink choices (14). To complement these results, Krukowski (17) reports that people continue to consume soft drinks because they consider them to have particular benefits, including taste and boosting energy. The present research confirms some of these findings. In particular, the participants of this study displayed a similar awareness of the potential negative health outcomes of drinking soft drinks. Nonetheless, this study went further to find that the source of the participants’ increased health awareness was primarily social media. The large volume of health-related information found on social media is playing a big part in driving greater health awareness, which, in turn, is encouraging people to think again about how many soft drinks they consume.

The problem identified is the association that has been found between poor nutritional outcomes, poor health, and excessive consumption of soft drinks (6, 7, 45). For example, research has revealed that excessive soft drink consumption is linked to a raised risk of diabetes (6, 45). Moreover, a meta-analysis of 88 research papers (45) examining the connection between health, nutrition, and soft drink consumption, found a connection between soft drink consumption, weight gain, and increased calorie intake (45). Additionally, the analysis revealed that consumers who drank soft drinks typically consumed less calcium and other vital nutrients through milk and other foods and suffered from a higher risk of diabetes and other health conditions (45).

Soft drink consumption’s high prevalence makes it an important health issue. Aljaadi et al. (12) conducted national cross-sectional research, including the analysis of secondary data, namely the 2021 Sharik Diet and Health National Survey (SDHNS), to determine weekly levels of soft and energy drink consumption by a representative group of Saudi Arabian adults. A total of 3,928 of the 5,194 individuals surveyed were used in the analysis and over two-thirds (67%) were found to consume soft drinks weekly (12). Other research (46) into calorie intake and patterns of drink consumption, examined the undergraduate student community in eight public university colleges in the Saudi Arabian city of Dammam. The study explored the students’ drink consumption on a daily and weekly basis (46). The study found that, on average, the undergraduates drank 1.5 liters of carbonated drinks weekly and 4.2 liters of sugar-sweetened drinks weekly. While the study (12) and the study (46) differ in terms of their participant group sizes and how outcomes were measured, the high prevalence of soft drink consumption found in both studies suggests that efforts should be made to reduce soft drink consumption.

According to Public Health England (47), the general public’s sugar consumption can be decreased using a range of interventions and strategies. One common approach is to target advertising and marketing campaigns on social media, television, and other media that can influence an individual’s sugar consumption (47). A further strategy is to target price promotions, which can encourage people to consume high-sugar beverages and food, by introducing taxes on high-sugar soft drinks (46). A third approach to lowering the public’s sugar intake is to reduce the amount of sugar contained in popular drinks and food by altering their recipes (47). Block et al. (15) identify further actions designed to reduce the consumption of soft drinks specifically. The authors argue that the consumption of soft drinks can be decreased through the use of programs based in schools. To illustrate, the soft drinks in school vending machines could be replaced with bottled water (48). Also, regulations governing soft drink sales in canteens in schools and soft drink consumption in schools more generally could be introduced (48). Block et al. (15) also recommend population-scale interventions such as interventions targeting the point of sale of soft drinks. Moreover, people involved in all the various sugar-consumption-related public health interventions have advocated for the imposition of a soft drink tax (49, 50).

### 4.1 Study implications

The results of this research have numerous implications. To begin, the importance of taste as a driver of soft drink consumption indicates that the issue of taste needs to be considered by any health interventions designed to reduce the consumption of soft drinks. Providing healthier drink alternatives with pleasant flavors may be necessary. Also, the important role played by habit regarding soft drink consumption shows that interventions seeking to change this consumption need to consider changing the behaviors around it to help people establish healthier dietary routines. Furthermore, the fact that the participants do not consider price when choosing to drink soft drinks indicates that interventions that implement price changes may be required. Finally, the effects of the environment and social contexts on consumption, for example, social events and restaurant dining, suggest that interventions need to focus on changing environment within these contexts. To illustrate, venues such as restaurants could provide healthier drink options that are promoted to consumers.

### 4.2 Study limitations

It is important to note that this study has a number of limitations. The data collection in this research relied on only one instrument: semi-structured interviews. Interviews are an important tool for gaining detailed insights into experiences and attitudes but they can sometimes only reveal part of the picture. To address this, future research done in this area could also use observation to collect data to complement the information gained through interviews and give a more complete picture of soft drink consumption. Finally, this research focused on identifying and exploring the factors that were impacting the participants’ consumption of soft drinks. This has resulted in important new insights into what shapes the behavior around soft drink consumption but it does not explore the participants’ opinions on any solutions that could help the participants to make healthier drink choices.

### 4.3 Future research direction

Future studies in this area may wish to explore the opinions of participants on the factors that influence their soft drink consumption and their suggestions on interventions that could help them make healthier choices. To achieve this, qualitative interviews could be conducted to gather data on participants’ opinions about interventions designed to help them choose healthier drink alternatives.

## 5 Conclusion

To conclude, this research highlights the value of pursuing a holistic approach to creating interventions designed to promote healthy drink choices and reduce the consumption of soft drinks. A holistic strategy should consider all five of the themes identified in this research, namely, taste, habit, price, environment and social context and health concerns. Addressing all of these factors simultaneously can be more effective. In particular, interventions should concentrate on creating healthier drink alternatives that come in enjoyable flavors, imposing sugar taxes to drive up the price of soft drinks, promoting the creation of healthier dietary routines and targeting contexts that encourage the consumption of soft drinks. A comprehensive strategy like this will help to reduce the consumption of soft drinks and encourage people to make healthier drink choices, supporting better general health and wellbeing among the population.

## Data Availability

The raw data supporting the conclusions of this article will be made available by the authors, without undue reservation.
